# 5′-Isoforms of miR-1246 Have Distinct Targets and Stronger Functional Impact Compared with Canonical miR-1246 in Colorectal Cancer Cells In Vitro

**DOI:** 10.3390/ijms25052808

**Published:** 2024-02-28

**Authors:** Rokas Lukosevicius, Gediminas Alzbutas, Greta Varkalaite, Violeta Salteniene, Deimante Tilinde, Simonas Juzenas, Ugne Kulokiene, Dainius Janciauskas, Lina Poskiene, Kestutis Adamonis, Gediminas Kiudelis, Juozas Kupcinskas, Jurgita Skieceviciene

**Affiliations:** 1 Institute for Digestive Research, Academy of Medicine, Lithuanian University of Health Sciences, LT-50161 Kaunas, Lithuania; rokas.lukosevicius@lsmuni.lt (R.L.); gediminas.alzbutas@lsmuni.lt (G.A.); greta.varkalaite@lsmuni.lt (G.V.); violeta.salteniene@lsmuni.lt (V.S.); deimante.valentelyte@lsmuni.lt (D.T.); simonas.juzenas@gmail.com (S.J.); ugne.kulokiene@lsmuni.lt (U.K.); juozas.kupcinskas@lsmuni.lt (J.K.); 2 Institute of Biotechnology, Life Science Centre, Vilnius University, LT-10257 Vilnius, Lithuania; 3 Department of Pathology, Medical Academy, Hospital of Lithuanian University of Health Sciences, LT-50161 Kaunas, Lithuania; dainius.janciauskas@lsmu.lt (D.J.); lina.poskiene@lsmu.lt (L.P.); 4 Department of Gastroenterology, Academy of Medicine, Lithuanian University of Health Sciences, LT-50161 Kaunas, Lithuania; kestutis.adamonis@kaunoklinikos.lt (K.A.); gediminas.kiudelis@lsmuni.lt (G.K.)

**Keywords:** CRC, miR-1246, 5′-isomiRs, targetome, 5′-isomiR functions in vitro

## Abstract

Colorectal cancer (CRC) is a multifactorial disease involving genetic and epigenetic factors, such as miRNAs. Sequencing-based studies have revealed that miRNAs have many isoforms (isomiRs) with modifications at the 3′- and 5′-ends or in the middle, resulting in distinct targetomes and, consequently, functions. In the present study, we aimed to evaluate the putative targets and functional role of miR-1246 and its two 5′-isoforms (ISO-miR-1246_a and ISO-miR-1246_G) in vitro. Commercial Caco-2 cells of CRC origin were analyzed for the expression of WT-miR-1246 and its 5′-isoforms using small RNA sequencing data, and the overabundance of the two miR-1246 isoforms was determined in cells. The transcriptome analysis of Caco-2 cells transfected with WT-miR-1246, ISO-miR-1246_G, and ISO-miR-1246_a indicated the minor overlap of the targetomes between the studied miRNA isoforms. Consequently, an enrichment analysis showed the involvement of the potential targets of the miR-1246 isoforms in distinct signaling pathways. Cancer-related pathways were predominantly more enriched in dysregulated genes in ISO-miR-1246_G and ISO-miR-1246_a, whereas cell cycle pathways were more enriched in WT-miR-1246. The functional analysis of WT-miR-1246 and its two 5′-isoforms revealed that the inhibition of any of these molecules had a tumor-suppressive role (reduced cell viability and migration and promotion of early cell apoptosis) in CRC cells. However, the 5′-isoforms had a stronger effect on viability compared with WT-miR-1246. To conclude, this research shows that WT-miR-1246 and its two 5′-isoforms have different targetomes and are involved in distinct signaling pathways but collectively play an important role in CRC pathogenesis.

## 1. Introduction

MicroRNAs (miRNAs), important regulatory molecules [1] involved in a number of pathological conditions (including cancer [2,3,4,5]), have been shown to have multiple isoforms (isomiRs) [6]. Although miRNAs are annotated and cataloged as single-defined sequences (termed canonical miRNAs (the most abundant sequence variant listed in the miRbase [7])), alternative processing (including alternative Drosha- and Dicer-mediated cleavage) and post-transcriptional modifications (including non-templated nucleotide addition and RNA editing) along the miRNA biogenesis pathway generate multiple isomiRs and thereby expand the regulatory repertoire of miRNA genes [6,8].

isomiRs have been shown to function similarly to canonical miRNAs since they actively associate both with the RISC and with translational machinery polysomes [6,9]. isomiRs with 5′-end variations are relatively rare compared to those with 3′-end variations. However, they have been shown to be important on a functional and pathophysiological level (including differential expression in cancer [10]). Due to the heterogeneity at the 5′-end, ‘seed-shifting’ might take place (e.g., in nucleotides 2–8 at the 5′-end), and, thereby, a change in target genes might occur [11,12,13,14,15]. However, the current knowledge regarding the targets of isomiRs and their functional roles remains limited.

Colorectal cancer (CRC) is currently the third most prevalent cancer type worldwide and is a leading cause of cancer-related mortality [16]. Despite the increasing availability of fecal occult blood tests and colonoscopy-based CRC screening programs, more precise methods for early, non-invasive disease detection are urgently needed [17]. A number of array-based, targeted, and high-throughput sequencing studies have shown the importance of miRNAs in CRC and have evaluated their suitability for diagnostic or prognostic purposes [18]. Recently, our group performed miRnome profiling in a colon adenoma–carcinoma sequence and identified miR-1246 to be deregulated in the early carcinogenesis [19]. Furthermore, we identified that ISO-miR-1246_a and ISO-miR-1246_G were more deregulated in CRC patients’ colon tissue compared with that of healthy controls (Supplementary Figure S1). Canonical miR-1246 (WT-miR-1246) is known to act as an oncogenic miRNA and targets genes such as *p53* [16], *CADM1* [20], *CCNG2* [16], and *THBS2* [16]. It is known that this miRNA participates in differentiation, invasion, and metastasis and promotes tumor growth and the chemoresistance of certain types of tumor cells [20,21,22]. However, there is no knowledge about the possible target genes or the functional relevance of miR-1246 isoforms in cancer, including CRC.

In this study, the miRNA profile (including the most common miR-1246 isoforms) of CRC cell lines was determined using small RNA-seq. The candidate target genes of canonical miR-1246 and its two 5′-isoforms were identified using RNA-seq and in silico target prediction analysis. Although the pathway enrichment analysis of deregulated target genes indicated that the three studied miR-1246 variants only partially shared signaling pathways, the functional analysis of the isoforms revealed that they all have a tumor-promoting role in CRC pathogenesis.

## 2. Results

### 2.1. 5′-Isoforms Dominate in miR-1246 Fraction and Have Different Targetomes

The miRNA-seq analysis of commercial CRC cell line Caco-2 was performed in order to check whether ISO-miR-1246_a and ISO-miR-1246_G could be detected in these particular cells. Within the framework of this study, the sequencing data indicated that WT-miR-1246 accounted only for a minor part (Cadco-2—31%) compared with ISO-miR-1246_a (Caco-2—51%) and ISO-miR-1246_G (Caco-2—17%) (Supplementary Figure S1).

**Figure 1:**
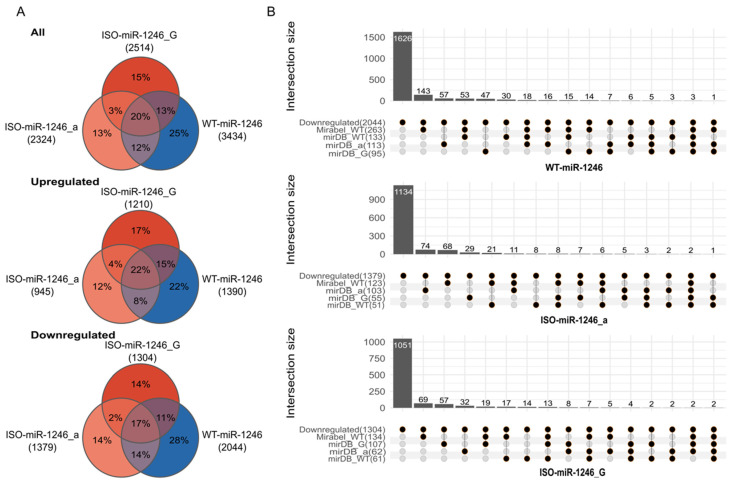
Differentially expressed genes and overlap of downregulated genes with predicted miRNA targets. (**A**)—A Venn diagram showing the number of differentially expressed (DE) genes between mimic treatment groups. Absolute values are indicated by the labels and relative values within each intersection. (**B**)—The number of predicted targets of WT-miR-1246 and the two 5′-isoforms, ISO-miR-1246_a and ISO-miR-1246_G, among the downregulated genes. The set labels indicate the data source (i.e., Mirabel_WT and miRDB labels; ISO-miR-1246_a—miRDB_a label; ISO-miR-1246_G—miRDB_G). The total count of the genes matching a particular set among the downregulated genes is indicated in parentheses next to the set label. The first column indicates the number of genes that are downregulated but are not among the predicted targets in any of the analyzed sets derived from the mirDB and Mirabel databases. The titles of the plots indicate the matching isomiR.

The transcriptome analysis revealed 3434 deregulated genes in Caco-2-transfected cells with a WT-miR-1246 mimic (59.52% downregulated; 40.48% upregulated), 2324 genes with an ISO-miR-1246_a mimic (59.34% downregulated; 40.66% upregulated), and 2514 genes with an ISO-miR-1246_G mimic (51.87% downregulated; 48.13% upregulated) (Figure 1A). The most impacted genes for the ISO-miR-1246_a, ISO-miR-1246_G, and WT-miR-1246 mimic treatment groups are presented in Supplementary Figures S5 and S6, as well as being listed with the complete gene enrichment analysis results in Supplementary File “All DE genes” at GEO under the accession number GSE220728. Thus, it is evident that significant differences between the three groups exist.

**Figure 2:**
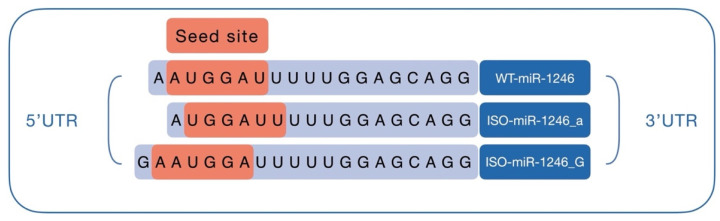
Illustration of seed sites of WT-miR-1246 and its two 5′-isoforms.

The seed sequences of WT-miR-1246, ISO-miR-1246_a, and I SO-miR-1246_G are different (Figure 2); therefore, they might have unique targetomes. This is clearly shown in Supplementary Figure S2. This figure shows the overlap of gene sets matching the predicted targets of WT-miR-1246 and its two isoforms. As can be seen in Supplementary Figure S2, the Mirabel database gives more sensitive predictions than MirDB—among all predictions for the mir-1246 isoforms, one-third (33%) are unique to Mirabel. Unfortunately, at the time of analysis, Mirabel predictions were available only for the wild-type variant. As can be seen in Supplementary Figure S2B, mirDB predicts a distinct set of genes for each isoform. Among all predicted genes for the three miR-1246 isoforms, only 14% are predicted to be targets of more than one isoform.

In silico predictions revealed that putative target sequences for the three miR-1246 isoforms were found in 20% of the downregulated genes (WT-miR-1246—418, ISO-miR-1246_a—245, and ISO-miR-1246_G—253) (Figure 1B). The majority of the putative targets in the downregulated genes were unique to each miRNA (WT-miR-1246—54%, ISO-miR-1246_a—30%, and ISO-miR-1246_G—23%). Only 1% of the targets in the downregulated genes (2–3 genes) were shared between the three isoforms (MiRabel and miRDB.org databases) (Figure 1B). The largest numbers of shared putative targets among the downregulated genes were between ISO-miR-1246_G and WT-miR-1246 (42 of 253 targets (16.6%)) and between WT-miR-1246 and ISO-miR-1246_a (49 of 418 targets (11.7%)). ISO-miR-1246_a and ISO-miR-1246_G shared approximately 5% of their targetomes among the downregulated genes.

### 2.2. Transcriptome-Based Targetome Analysis Reveals Differential Enrichment of Signaling Pathways between WT-miR-1246 and Its 5′-Isoforms

**Figure 3:**
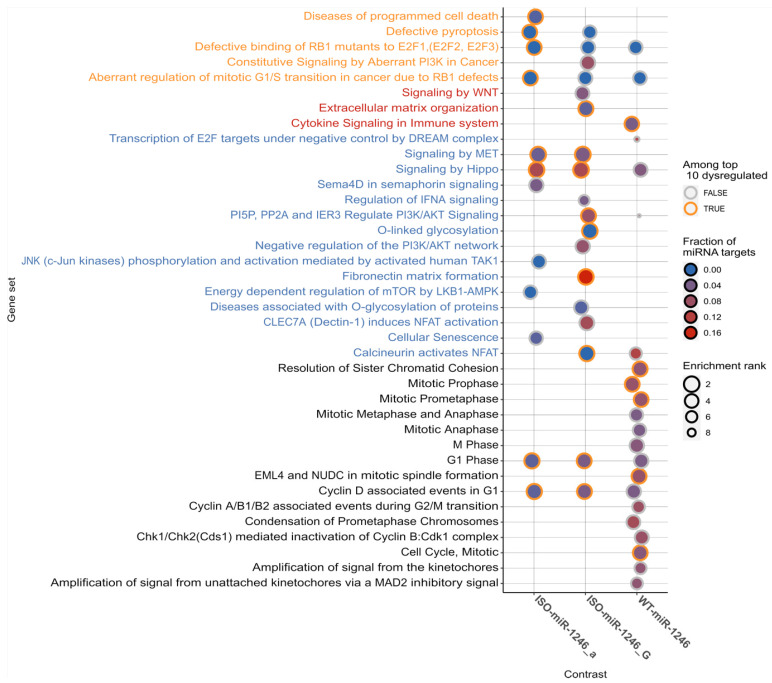
The most enriched pathways (Reactome database). For each mimic group, the most enriched pathways were chosen for visualization. The pathways included in a Reactome cancer-related pathway list are denoted by the orange color; red denotes pathways that match the subpathways of KEGG cancer pathways (hsa05200); blue denotes other potential cancer-related pathways; black denotes potential cell-cycle-/division-related pathways. The circle size is inversely proportional to the pathway enrichment rank—the larger the circle, the more significant the enrichment. If a gene set is among the top 10 gene sets for a particular mimic group, the border of the corresponding circle is orange. The fill color denotes the fraction of genes that were indicated to be targeted by WT-miR1246 or its two isoforms by any of the used databases.

The pathway enrichment analysis revealed that the dysregulated genes of the studied WT-miR-1246 and its 5′-isoforms were differentially involved in the cancer-related and cell cycle pathways (Figure 3 and Supplementary Figures S8 and S9 (Reactome database analysis) and Supplementary Figures S10 and S11 (KEGG database analysis)). Cancer-related pathways were more enriched in dysregulated genes in the ISO-miR-1246_a and ISO-miR-1246_G cases (e.g., “signaling by MET” pathway (enrichment rank ISO-miR-1246_G—1.7, *p* = 5.47 × 10^−8^; ISO-miR-1246_a—1.8, *p* = 4.23 × 10^−11^), “signaling by Hippo” pathway (enrichment rank ISO-miR-1246_G – 1.8, *p* = 8.57 × 10^−8^; ISO-miR-1246_a—1.9, *p* = 3.96 × 10^−8^), “PI5P, PP2A, and IER3 regulate PI3K/AKT signaling” pathway (enrichment rank ISO-miR-1246_G—2.7, *p* = 1.24 × 10^−4^) etc.). These dysregulated pathways stand out as they have a large number of genes with expected miRNA targets in silico (e.g., “signaling by Hippo pathway”: *LATS2* (ISO-miR-1246_G: log_2_FC = −0.62, *p* = 1.28 × 10^−47^; ISO-miR-1246_a: log_2_FC = −0.19, *p* = 4.0 × 10^−6^; WT-miR-1246: log_2_FC = −0.49, *p* = 1.06 × 10^−30^); “PI5P, PP2A, and IER3 regulate PI3K/AKT signaling” pathway: *EREG* (ISO-miR-1246_G: log_2_FC = −0.95, *p* = 8.94 × 10^−23^; WT-miR-1246: log_2_FC = −0.67, *p* = 2.03 × 10^−12^), *PPP2CB* (ISO-miR-1246_G: log_2_FC = −0.79, *p* = 1.76 × 10^−48^; WT-miR-1246: log_2_FC = −0.40, *p* = 2.26 × 10^−13^), etc.) (Figure 3 and Supplementary Figure S8). On the other hand, the unique enrichment of signaling pathways was also determined. The cancer-related pathway “signaling by WNT” was significantly dysregulated only in the ISO-miR-1246_G case (enrichment rank 3.7, *p* = 2.68 × 10^−4^). This pathway had a few dysregulated predicted targets for ISO-miR-1246_G (*PPP2CB*, *PSMA5*, *RBBP5*). The “fibronectin matrix formation” and “extracellular matrix organization” pathways were also significantly dysregulated but only in the ISO-miR-1246_G case (enrichment ranks 1.9, *p* = 3.14 × 10^−6^ and 2.6, *p* = 1.06 × 10^−4^, respectively). “Diseases of programmed cell death”, “JNK (c-Jun kinase) phosphorylation and activation mediated by activated human TAK1, Sema4D in semaphorin signaling”, and “energy-dependent regulation of mTOR by LKB1-AMPK” were uniquely enriched in the ISO-miR-1246_a case (enrichment rank 2.8, *p* = 1.79 × 10^−4^; 3.7, *p* = 3.99 × 10^−5^; 3.4, *p* = 3.87 × 10^−4^; 4.1, *p* = 3.76 × 10^−4^, respectively). As a component of “Sema4D in semaphorin signaling”, *LIMK2* is a potential target gene of ISO-miR-1246_a and was dysregulated (log2FC = −0.31, *p* = 1.29 × 10^−24^) after transfection with this isomiR.

The pathway enrichment analysis also revealed some similarities in cell-cycle-related pathways between the three isomiR groups. “Cyclin D associated events in G1” and “G1 phase” were found to be enriched in WT-miR-1246 (enrichment rank 3.3, *p* = 1.49 × 10^−5^; 3.3, *p* = 1.49× 10^−5^, respectively), ISO-miR-1246_a (enrichment rank 2.3, *p* = 4.99 × 10^−5^; 2.3, *p* = 4.99 × 10^−5^, respectively), and ISO-miR-1246_G (enrichment rank 2.7, *p* = 1.24× 10^−4^; 2.7, *p* = 1.24× 10^−4^, respectively). However, WT-miR-1246 was found to be more enriched in cell-cycle-related pathways and contained more dysregulated genes (Figure 3 and Supplementary Figure S9). Among the most dysregulated pathways for WT-miR-1246, we found pathways such as “resolution of sister chromatid cohesion” (enrichment rank 2.6, *p* = 2.47 × 10^−6^), “mitotic prophase” (enrichment rank 2.8, *p* = 4.23 × 10^−6^), and “cell cycle, mitotic” (enrichment rank 2.9, *p* = 4.77 × 10^−6^). These pathways were found to be dysregulated only in WT-miR-1246. The majority of the highly impacted genes in these pathways were found in the pathway “cell cycle, mitotic” (Supplementary Figure S9). Among the eight most downregulated genes in these pathways, five genes were predicted to be potential targets of WT-miR-1246: *CDC25A* (log_2_FC = −1.11, *p* = 2.04 × 10^−48^), *PPP2CB* (log_2_FC = −0.40, *p* = 2.3 × 10^−13^), *DYNC1I1* (log_2_FC = −0.33, *p* = 3.2 × 10^−6^), *TUBGCP3* (log_2_FC = −0.31, *p* = 2.1 × 10^−16^), and *NUP50* (log_2_FC = −0.29, *p* = 7.1 × 10^−15^).

Details of the pathway enrichment can be found in the reactome_impact.html file at GEO under the accession number GSE220728, whereas information on the log_2_FCs and genes can be found in the Supplementary File “All DE Genes” at GEO under the accession number GSE220728.

### 2.3. Inhibition of WT-miR-1246 and Its 5′-Isoforms Reduces Cell Viability

**Figure 4:**
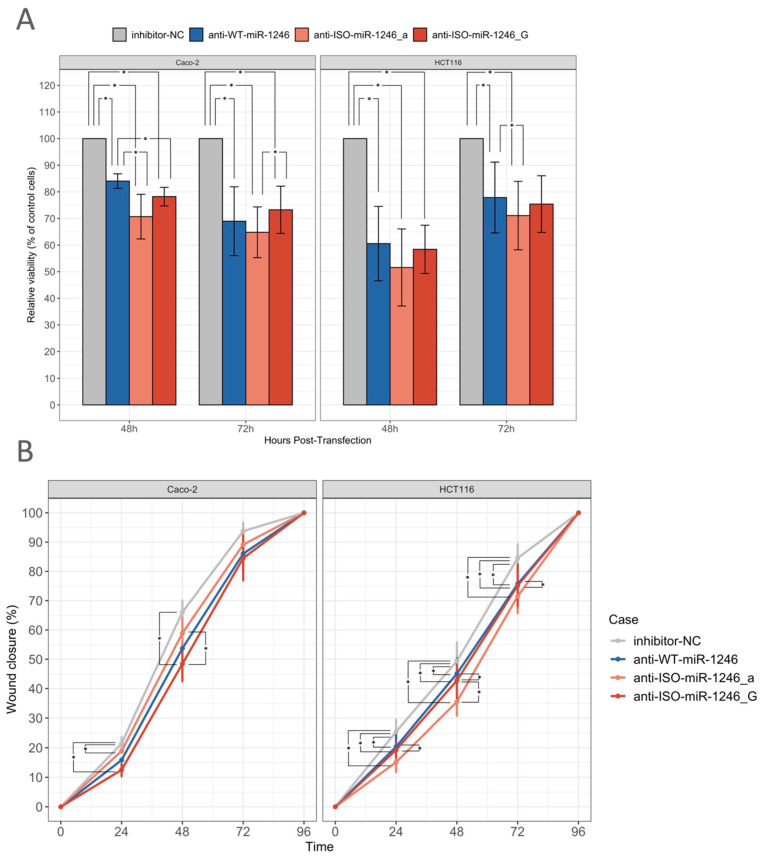
(**A**)—Barplot represents changes in cell viability 48 h and 72 h after transfection with miRNA inhibitors (anti-WT-miR-1246, anti-ISO-miR-1246_a, anti-ISO-miR-1246_G) relative to cells transfected with negative control inhibitor (inhibitor-NC). (**B**)—Line plot shows cell migration rates, represented as a percentage of the covered gap area, measured 0–96 h after transfection with miRNA inhibitors. Data are presented as the mean (three or more independent experiments) ± standard error (SE); *—data are statistically significant when *p* < 0.05.

To evaluate the impact of miR-WT-1246 and its two 5′-isoforms on CRC cell viability, the MTT assay was performed, analyzing Caco-2 and HCT116 cells after transfection with the respective miRNA inhibitors. The inhibitors of WT-miR-1246 and its 5′-isoforms significantly reduced the cell viability in Caco-2 cells at 48 h (anti-WT-miR-1246 by 16%, *p* = 0.03; anti-ISO-miR-1246_a by 29%, *p* = 0.03; anti-ISO-miR-1246_G by 22%, *p* = 0.01) and 72 h post-transfection (anti-WT-miR-1246 by 31%, *p* = 0.02; anti-ISO-miR-1246_a by 35%, *p* = 0.03; anti-ISO-miR-1246_G by 27%, *p* = 0.03) compared to the cells transfected with the negative control inhibitor. Experiments in HCT116 cells showed similar results. Significantly reduced cell viability was observed 48 h (anti-WT-miR-1246 by 39%, *p* = 0.01; anti-ISO-miR-1246_a by 48%, *p* = 0.01; anti-ISO-miR-1246_G by 42%, *p* = 0.02) and 72 h after transfection (anti-WT-miR-1246 by 22%, *p* = 0.03; anti-ISO-miR-1246_a by 23%, *p* = 0.02; anti-ISO-miR-1246_G by 25%, *p* = 0.06) when comparing cells transfected with inhibitors of WT-miR-1246 and its 5′-isoforms and the negative control inhibitor (Figure 4A). Notably, comparing cell viability within the isomiR groups, both anti-ISO-miR-1246_a and anti-ISO-miR-1246_G had greater effects on cell viability compared with anti-WT-miR-1246 in Caco-2 cells at 48 h post-transfection (by 14%, *p* = 0.04 and 6%, *p* = 0.004, respectively), while, in HCT116, only anti-ISO-miR-1246_a showed a stronger effect at 72 h post-transfection (by 7%, *p* = 0.003). Interestingly, anti-ISO-miR-1246 showed a significantly stronger effect in Caco-2 cells at 72 h post-transfection than anti-miR-1246_G (by 8%, *p* = 0.02).

### 2.4. Inhibition of WT-miR-1246 and Its 5′-Isoforms Slows Gap Closure in Wound Healing Assay 

An effect on the cell migration rate was observed in both HTC116 and Caco-2 cells after transfection with the inhibitors of WT-miR-1246 and its 5′-isoforms compared to cells transfected with the negative control inhibitor. As a result, the inhibition of WT-miR-1246, ISO-miR-1246_a, and ISO-miR-1246_G significantly slowed gap closure at 24 h (5%, *p* = 5.77 × 10^−4^; 10%, *p* = 3.98 × 10^−3^; 6% *p* = 0.045, respectively), 48 h (4%, *p* = 0.035; 14%, *p* = 0.013; 7%, *p* = 4.82 × 10^−3^), and 72 h post-transfection (8%, *p* = 0.036; 13%, *p* = 0.01) compared to the negative control inhibitor in HTC116 cells. However, only anti-ISO-miR-1246_G had a significant effect on the cell migration rate in Caco-2 cells at 24 h (by 9%, *p* = 3.15 × 10^−5^) and 48 h post-transfection (by 18%, *p* = 0.015), while anti-ISO-miR-1246_a slowed gap closure only at 48 h post-transfection (by 7%, *p* = 0.033) (Figure 4B). Interestingly, HCT116 cell treatment with anti-ISO-miR-1246_a showed a significantly stronger effect compared to anti-WT-miR-1246 treatment at all time points (24 h—5%, *p* = 0.03; 48 h—9%, *p* = 0.01; 72 h—4%, *p* = 0.03). Moreover, anti-ISO-miR-1246_a showed a significantly stronger effect at 48 h post-transfection compared to anti-ISO-miR-1246_G in HCT116 (4%, *p* = 0.03). However, Caco-2 cell treatment with anti-ISO-miR-1246_G had a greater effect than that with anti-miR-1246_a at 48 h post-transfection (5%, *p* = 0.007).

### 2.5. Inhibition of ISO-miR-1246_G Affects Rates of Early Apoptosis in SW620 Cells

The flow-cytometry-based Annexin V Pacific Blue/PI assay was used for the evaluation of early apoptosis and death in the cell culture after transfection with the WT-miR-1246 and its two isomiR inhibitors. Annexin V Pacific Blue-positive cells were considered to be early apoptotic, and Annexin V Pacific Blue/PI-positive cells were considered to be late apoptotic/necrotic cells. The inhibition of WT-miR-1246, ISO-miR-1246_a, and ISO-miR-1246_G had no significant effect on the apoptosis rates of HCT116 and SW620 cells. However, there was a statistically significant difference in the fraction of early apoptotic cells when comparing anti-ISO-miR-1246_G-transfected SW620 cells to the inhibitor-NC group (*p* = 4.25 × 10^−3^) (Supplementary Figure S7A).

### 2.6. Inhibition of WT-miR-1246 and Its Isoforms Does Not Affect Cell Proliferation

The effect of WT-miR-1246 and its 5′ -isoforms on the modulation of cells’ proliferation was analyzed using CFSE and flow cytometry. SW620 and HCT116 cells were reverse-transfected with anti-WT-miR-1246, anti-ISO-miR-1246_a, and anti-ISO-miR-1246_G, stained and analyzed using flow cytometry after 96 h of incubation. Cell proliferation showed a tendency to be slowed in the HCT116 cell line after treatment with WT-miR-1246, ISO-miR-1246_a, and ISO-miR-1246_G by 9.1% (*p* = 0.53), 34.6% (*p* = 0.2), and 21.3% (*p* = 0.4), respectively. In the SW620 cells transfected with inhibitors of the studied miRNAs, the effect was minor: WT-miR-1246—13.9% *p* = 0.45; ISO-miR-1246_a—4% *p* = 0.63; and ISO-miR-1246_G—5.9% *p* = 0.58. However, significance was not reached in either of the cell lines (Supplementary Figure S7B).

### 2.7. Inhibition of WT-miR-1246 and Its 5′-Isoforms Does Not Affect Colony Formation Rate

To examine the role of WT-miR-1246 and its 5′-isoforms in CRC cells’ division capabilities, a colony formation assay was performed. The number of colonies was reduced in both SW620 (anti-WT-miR-1246 by 28%; anti-ISO-miR-1246_a by 22%) and HCT116 cells (anti-WT-miR-1246 by 51%; anti-ISO-miR-1246_a by 45%) after the inhibition of WT-miR-1246 and ISO-miR-1246_a, compared to cells transfected with the negative control inhibitor, while the inhibition of ISO-miR-1246_G reduced the colony number only in HCT116 cells, by 28%. However, the results for the two cell lines and all miRNAs were not statistically significant (Supplementary Figure S7C).

## 3. Discussion

The deregulation of miRNAs occurs in almost all major types of cancer, including CRC, and is associated with disease initiation, progression, and metastasis [2,3,4,5]. Previous studies have revealed that canonical miRNA sequences can have various isoforms (isomiRs), which may present distinct expression patterns and/or different target genes [1]. This indicates that the regulatory mechanisms underlying the function of miRNAs in oncogenesis are much more complex than previously believed. To date, isomiRs have been comprehensively studied only in breast cancer [14], melanoma [23], and gastric cancer [24], and no previous study on CRC has been performed in vitro.

Within the framework of this study, we chose to analyze the 5′-isomiRs of miR-1246, which have been reported by our group to be deregulated in the early stages of colorectal carcinogenesis [19]. Although a considerable amount of functional (basic and clinical) and regulatory information is known about the canonical oncogenic miR-1246, there is no knowledge of the possible target genes of its isoforms or their functional roles. We chose to analyze the isoforms of the 5′-end, as they have an altered seed sequence compared to the canonical microRNA (Figure 2) and, therefore, are expected to display an altered target site selection [25].

Firstly, we performed small-RNAseq on Caco-2 commercial CRC cells and identified that ISO-miR-1246_a (approximately 51%) and ISO-miR-1246_G (approximately 17%) were the most common isoforms, whereas WT-miR-1246 accounted for approximately 31%. These findings are in line with the data from different colon tissue samples [19,26]. Moreover, the significant upregulation of ISO-miR-1246_a and ISO-miR-1246_G in CRC and advanced adenoma has been shown in previous studies [26], thereby supporting the possible involvement of miR-1246 isoforms in carcinogenesis.

Secondly, to obtain experimental information about the targetomes of the studied miR-1246 isoforms, we performed Poly(A) RNA-seq on the Caco-2 cell line after transfection with mimics of WT-miR-1246, ISO-miR-1246_a, and ISO-miR-1246_G. There is a limited number of studies analyzing the targetomes of miRNA isoforms. Some authors argue that canonical miRNAs and their 5′-isomiRs generally have the same or similar gene targets and, consequently, common biological pathways [9,13]. However, nucleotide changes at the 5’-end of the miRNA sequence have been shown to cause altered binding to the mRNA sequence, which may lead to different target genes [6,14,27]. A study conducted by van der Kwast et al. showed that even the in-silico-predicted targets of wild-type and 5′-isoforms had only a minor overlap [28]. These results support our findings, which showed that the majority of the target genes were unique to WT-miR-1246 and two of its isoforms (only 1% of targets were shared between the miR-1246 and its two 5′-isoforms) (Supplementary Figure S2). This indicates that the 5′ sequence variants of miR-1246 significantly change the impacted gene sets. Moreover, the pathway enrichment analysis indicated that genes deregulated by WT-miR-1246 and its 5′-isoforms are involved in different signaling pathways, which was also shown in the van der Kwast et al. study [28].

Based on the pathway enrichment analysis, we observed that the pathways participating in canonical cancer-related processes, such as proliferation, motility, and invasion, were putatively impacted by all of the miR-1246 isoforms. The control of these canonical cancer-related processes by miRNAs, including WT-miR-1246, is well established [29]. However, more of the enriched pathways were found within the isoform groups. The Hippo, PI3K/AKT, and tumor microenvironment signaling pathways, responsible for cell proliferation, migration, and invasion, were more enriched in differentially expressed predicted target genes (*LATS2*, *NRAS*, *PPP2CB*, etc.) in cells transfected with mimics of ISO-miR-1246_a and/or ISO-miR-1246_G. 

Hippo signaling plays a critical role in modulating cell proliferation and has been demonstrated to contribute to the progression of various malignant diseases, including CRC [30]. Our analysis showed that the expression of the major genes of this pathway, *LATS2* (downregulated more than 1.5 times), *WWTR1* (upregulated 1.33 times), and *STK3* (downregulated 1.2 times), were affected after transfection with WT-miR-1246 and its two isoforms. In silico prediction indicated that both ISO-miR-1246_G (8-mer and 7-mer binding sites) and WT-miR-1246 (7-mer binding site) have direct binding sites in *LATS2*. The diminished expression of *LATS2* results in genetic instability and the loss of contact growth inhibition (i.e., high cell density) [31,32], while ectopic expression promotes apoptosis [33,34]. The deregulation of *LATS2* has been previously associated with a poor CRC prognosis. *LATS2* expression has been shown to be lower in CRC tissue compared to adjacent tissue and even lower in metastatic tumors compared to primary ones. The loss of *LATS2* switches natural replicative cell senescence to malignant transformation [35]. Previous studies have indicated that miR-103a-3p [36], miR-31-5p [37], and miR-492 [38] are directly involved in the regulation of *LATS2*, and the knockdown/downregulation of these miRNAs inhibited cell proliferation. Therefore, our data indicate that ISO-miR-1246_G and WT-miR-1246 are involved in the regulation of the Hippo pathway, including components of the YAP/WWTR1-induced feedback mechanism.

The PI3K/Akt/mTOR pathway is one of the main signaling cascades and is closely linked with a number of essential pathways, such as Ras/Raf/MEK/ERK, mTOR, and WNT, involved in the regulation of cell survival and proliferation [39]. The deregulation of this pathway has been reported in many solid tumors, including CRC (60–70% of cases) [40,41]. After transfection with ISO-miR-1246_G, ISO-miR-1246_a, and/or WT-miR-1246, the most deregulated genes in this pathway were *EREG* (downregulated 1.9 times), *PPP2CB* (downregulated 1.7 times), *MET* (upregulated 1.6 times), *PDGFRA* (downregulated 1.6 times), and *PIP4K2A* (upregulated 1.4 times). Furthermore, the performed gene silencer analysis (to test whether gene expression was related to the suppression of the matching silencer; for more details, see Supplementary Figure S12) identified that transfection with all miR-1246 isoforms resulted in a number of deregulated silencers and their targets within the PI3K/Akt pathway (*MET/CAV1* (silencer target of WT-miR-1246), *FARP2/HDLBP* (silencer target of WT-miR-1246), *SAMD5/STXBP5* (silencer target of WT-miR-1246), *NRAS/SIKE1* (silencer target of ISO-miR-1246_G), and *RFK/PCSK5* (silencer target of ISO-miR-1246_a)). The in silico analysis indicated that the two most downregulated genes of this pathway—*EREG* and *PPP2CB*—had possible binding sites in ISO-miR-1246_G (*EREG*: two 7-mer binding sites; *PPP2CB*: 8-mer binding site) and WT-miR-1246 (*EREG*: 7-mer binding site; *PPP2CB*: 7-mer binding site). The *PPP2CB* gene encodes a catalytic domain for protein phosphatase 2A (PP2A). PP2A is one of the major Ser/Thr phosphatases in eukaryotic cells [41], and the decreased activity of this protein (i.e., acting as a tumor suppressor) has been reported in many types of cancer, including CRC [42]. This phosphatase has been the focus of CRC therapy development, as previous clinical studies have indicated that PP2A is an independent prognostic factor for CRC [43]. The downregulation of *PPP2CB* is associated with a worse prognosis and overall survival for CRC [44]. A recently published study by Narayan et al. [44] provided evidence that the upregulation of PP2A and downregulation of the PI3K/Akt pathway members (AKT1, mTOR, 4EBP1, and p21) can be beneficial for the management of patients with CRC. A study by Bott et al. indicated that WT-miR-1246 directly targets *PPP2CB* in breast cancer cells [45]. Epiregulin (EREG) is an activator of the PI3K/AKT pathway, directly binding to the extracellular domain of the EGF receptor [46]. This protein is ubiquitously upregulated in many types of cancer [47,48] and has been indicated as a biomarker to predict the clinical benefit of cetuximab in patients with metastatic CRC [49]. Previous studies have indicated that miR-186-3p [50] and miR-215-5p [51] are directly involved in the regulation of *EREG*, and the upregulation of these miRNAs plays a functional role in controlling tumorigenesis (cell survival and cell proliferation). Therefore, our study is the first indicating the regulatory association between miR-1246, *PPP2CB*, and *EREG* in the CRC setting, and future studies analyzing the direct regulatory properties of this miRNA in the PI3K/Akt pathway in CRC would be of great interest.

The pathways responsible for the cell cycle were more enriched in WT-miR-1246 (mitotic prophase, cell cycle, mitotic, resolution of sister chromatid cohesion, etc.). However, the cell cycle control pathways (defective binding of RB1 mutants to E2F1, aberrant regulation of mitotic G1/S transition in cancer due to RB1 defects) and pathways responsible for the cell G1 phase (Cyclin-D-associated events in G1, G1 phase) were identified among the most deregulated pathways in cells transfected with ISO-miR-1246_a and ISO-miR-1246_G. The control of cell proliferation by miRNAs is well established, and tumor suppressor miRNAs induce cell cycle arrest by downregulating multiple components of the cell cycle machinery [52]. In our study, the most deregulated genes in these cell-cycle-related pathways were *CDC25A* (downregulated by WT-mir-1246 more than two times), *PPP2CB* (downregulated by ISO-miR-1246_G 1.7 times), *CCND3* (upregulated by all isoforms more than two times), and *E2F2* (upregulated by all isoforms 1.7 times). The most downregulated gene among the pathways related to the cell cycle was *CCD25A*, a known oncogene involved in tumor initiation and progression that correlates with poor patient prognoses [53]. WT-miR-1246 may have an 8-mer binding site in this gene, although this has not been confirmed in the laboratory setting. However, due to the oncogenic nature of both molecules, the exact regulatory mechanism and causal relationship must be proven in future studies. The second most downregulated gene is *PPP2CB*. It is a catalytic subunit of protein phosphatase 2A (PP2A) that plays a critical role in regulating the reorganization of cellular structures during mitosis [54]. The role of *PPP2CB* has been discussed in detail above in regard to the PI3K/Akt/mTOR pathway. A previous study indicated that miR-1246 might be involved in the regulation of the cell cycle [55]; therefore, our study provides additional evidence for the importance of this miRNA and its isoforms in the control of these important pathways.

In addition, we performed a functional analysis (i.e., tested the viability, migration, proliferation, and apoptosis) of miR-1246 and its isoforms by implementing loss-of-function experiments in CRC cell cultures. WT-miR-1246 is already known to play an oncogenic role by promoting tumor angiogenesis, growth, migration, invasion, and metastasis in many types of cancer, including CRC [20,21,56,57,58]. Our results are in agreement with the mentioned reports and show that the inhibition of WT-miR-1246 reduced the cell viability, migration rates, and apoptosis in CRC, confirming the oncogenic function of miR-1246. Nevertheless, the inhibition of ISO-miR-1246_a and ISO-miR-1246_G in our study had the same effect as the inhibition of WT-miR-1246 in the apoptosis and migration tests. Interestingly, the inhibition of ISO-miR-1246_a and ISO-miR-1246_G showed a stronger effect on viability compared to the wild-type miRNA, suggesting that these 5′-isomiRs might play the same oncogenic role in CRC. The stronger isoform effect in the migration test corresponded to our pathway enrichment data analysis, where we identified stronger effects of ISO-miR-1246_a and ISO-miR-1246_G in the Hippo pathway, which is known to be related to cell proliferation, with an enrichment compared to the wild-type miRNA.

This study has limitations that should be acknowledged. Firstly, the origin of miR-1246 is often questioned in the miR research field. Yi-Fan Xu et al. [58] reported that miR-1246 is an RNU2-1 degradation product. However, to address the raised concern, it is essential to examine whether the miRNA operates within Ago complexes, recognizing that the ARGONAUTE (AGO) protein family acts as the catalytic core of the silencing complex, guided to its specific RNA targets by sequence-specific small RNA [59]. In this context, we analyzed the publicly available data on immunoprecipitation with AGO antibodies and miRNA-seq in the data from BioProject PRJDB2698 [60] and BioProject PRJNA840916 [61]. Based on the analysis of the datasets, we found that miR-1246 was more abundant when immunoprecipitation was performed using the AGO1 (data from two studies, Supplementary Figure S11A) and AGO2 (data from one study, Supplementary Figure S11B) antibodies. For more details, please refer to “*Analysis of immunoprecipitation data from other studies*” in Supplementary section. Based on the analysis of the three datasets, we suggest that miRNA-1246 forms complexes with AGO proteins and acts as a miRNA. Furthermore, in our data, we found that the targets of this miRNA were enriched among the downregulated genes after transfection with mimics (Supplementary Figure S12). Moreover, our data showed the great importance of the ISO-miR-1246_G variant, which has the additional 5′ “G” that is not present in the sequence of RNU2-1. All of this evidence allows us to presume that miR-1246 and its two 5′-isomiRs act as miRNAs. Secondly, the non-sequencing methods for isomiR gene expression, e.g., dumbbell PCR in the Shigematsu et al. [62] methodology, are not accurate and lack specificity, leaving sequencing approaches the only option for isomiR detection. While the authors of the dumbbell PCR methodology stated that it detects 5′ and 3′ modifications of miR-16, they did not compare this method with existing qPCR assays with reference to miR-16.

In conclusion, we found that the 5′-isomiRs of miR-1246 (i.e., ISO-miR-1246_a and ISO-miR-1246_G) were more abundant than the wild-type miR-1246 itself in colon tissue samples (healthy and CRC) and in colorectal cancer cell lines. The transcriptome analysis revealed that the putative target genes between WT-miR-1246 and its 5′-isoforms overlapped by less than 1%. The signaling pathway enrichment analysis revealed that there was only a partial overlap between the miRNAs under study. The functional analysis of miR-1246 and its 5′-isoforms showed similar tumor-suppressive functions in the CRC cell lines; however, both isoforms showed stronger effects.

## 4. Materials and Methods

### 4.1. Cell Cultures

The human colorectal adenocarcinoma Caco-2 (HTB-37™, ATCC^®^, Manassas, VA, USA), SW620 (CCL-227™, ATCC^®^, Manassas, VA, USA), and HTC116 (CCL-247™, ATCC^®^, Manassas, VA, USA) cell lines were cultured in Ham’s F-12K (Kaighn’s) Medium (Gibco, Waltham, MA, USA), Leibovitz’s L-15 Medium (Gibco, Waltham, VA, USA), and McCoy’s 5a Medium Modified (Gibco, Waltham, MA, USA), respectively. All media were supplemented with fetal bovine serum (20% for Caco-2 cells and 10% for SW620, HTC116 (Gibco, Waltham, MA, USA)) and 1% penicillin/streptomycin (100 U/mL penicillin and 100 mg/mL streptomycin, Corning, New York, NY, USA). All cell lines were cultured at 37 °C in 5% CO_2_.

### 4.2. Transfection of Cell Cultures

The reverse transfection of ISO-miR-1246_G and ISO-miR-1246_a with the Custom mirVana™ miRNA Inhibitor (Invitrogen™, Carlsbad, CA, USA) and/or Custom mirVana™ miRNA Mimic (Invitrogen™, Carlsbad, CA, USA), and that of WT-miR-1246 with miRNA Mimics & Inhibitors (assay ID PM13182), as well as negative control #1 (Invitrogen™, Carlsbad, CA, USA), was performed using Lipofectamine 3000 reagent (Invitrogen™, Carlsbad, CA, USA). A final concentration of 100 pmol/mL per mimic and inhibitor was used for all studied miRNAs and the negative control. For the functional cell assays (MTT, wound healing, etc.), inhibitors of the studied miRNAs were used, whereas for transcriptome sequencing, miRNA mimics were used.

### 4.3. RNA Isolation, Preparation of RNA Libraries, and Next-Generation Sequencing

Total RNA from colorectal adenocarcinoma cells Caco-2 (n = 2) (for cell line miRNA profiling) and transfected Caco-2 cells (5 replicates per isoform (total n = 20)) (for transcriptome sequencing) was extracted using the miRNeasy Mini Kit (Qiagen, MD, USA) 48 h post-transfection, according to the manufacturer’s recommendations.

Polyadenylated RNA sequencing libraries were generated with 1 µg of total RNA input per sample, applying a standard Illumina TrueSeq Stranded mRNA (Illumina, CA, USA) protocol. The TapeStation 2200 system (Agilent, Santa Klara, CA, USA) and a Qubit 4 fluorimeter (Thermo Fisher Scientific, Waltham, MA, USA) were used for the quality and yield assessment of the sequencing libraries. The RNA libraries were sequenced (2 × 100 bp paired-end reads) on the NovaSeq 6000 (Illumina, San Diego, CA, USA) next-generation sequencing (NGS) platform.

Small RNA sequencing was performed for Caco-2 cells. Sequencing libraries were constructed using the TruSeq Small RNA Sample Preparation Kit (Illumina, San Diego, CA, USA) and sequenced on the HiSeq2500 (Illumina, San Diego, CA, USA) NGS platform. A detailed description of the small RNA sequencing protocol is provided in our previous article [19].

### 4.4. Poly(A) RNA Sequencing and Small RNA Sequencing Data Analysis

The bioinformatics analysis of the small RNA sequencing data involved the following steps: (i) 3′-end adapter, low-quality base (<Q20), and short read (<18 nt) removal using cutadapt [63]; (ii) the mapping and annotation of miRNA sequences using mirAligner [64] and miRbase(REF) v21 as a reference; (iii) the quantification of mature miRNA isoforms using the isomiRs R package [65] with default parameters. The relative abundance for each isoform in each sample was estimated by dividing the normalized count of a given isoform (numerator) by the sum of the total normalized count, which was mapped to the same miRNA arm (denominator).

The whole-transcriptome NGS-generated raw sequencing reads (.fastq) were subjected to read quality trimming and adapter sequence removal using the BBduk program from the BBtools v38.87 package [66]. Reads longer than 75 nt and with more than two counts/samples were used for further analysis. Processed reads were aligned to the human reference genome GRCh38.p13 using STAR v2.7.9 Gencode v34 annotations [67]. Counts per gene were detected using Verse v0.1.5. ComBat-seq was applied for batch effect (cell culture passage number and condition) removal. Further analysis was performed using DESeq2 v1.32.0 [68]. The log_2_FCs were shrunken using the ‘ashr’ tool in R (implementing an Empirical Bayes approach and false discovery rate (FDR corrected *p* < 0.05) estimation) [69]. Differentially expressed (DE) genes were detected using five groups, comparing them with the negative control or WT-miR-1246 mimic. The abbreviations of the five comparison groups are explained in the Supplementary Materials, “DE genes comparison”.

### 4.5. In Silico Target Prediction of microRNAs

Putative targetomes of the reference WT-miR-1246 and the two 5′-isoforms (ISO-miR-1246_G and ISO-miR-1246-a) were retrieved from the publicly available database miRDB.org v6.0 [70]. Additionally, a potential targetome for the reference WT-miR-1246 was retrieved from miRabel (as available on 16 June 2021), known to be one of the most accurate and specific tools [71]. miRabel works only with annotated canonical miRNAs and not with isoforms. The hits identified by miRabel and miRDB.org were filtered to retain only those with *p* ≤ 0.05.

### 4.6. Pathway Enrichment Analysis

The shrunken log_2_FC values were subjected to enrichment analysis using the Gene Graph Enrichment Analysis (GGEA) [72] and PathNet [73] methods, implemented in EnrichmentBrowser (RELEASE_3_13) [74]. This package was also used for the integration of the results produced by the two programs. The gene sets of the pathways were obtained from the Reactome and KEGG databases. The final ranking of pathways was based on the combined *p*-value rankings of the PathNet and GGEA results (which resulted in continuous rank values), and only those with significant dysregulation (FDR corrected *p*-value < 0.05) indicated by both programs were considered for further analysis and interpretation (full explanation in Supplementary Materials). The joined *p*-value combining the results of the two programs was calculated via the sum of logs (Fisher’s) method using the meta package [75]. The rationale behind selecting the two programs is given in the Supplementary Materials (Supplementary Figures S3 and S4).

### 4.7. MTT Assay

The MTT assay was used for the assessment of the metabolic activity of the cells, as it reflects the quantity of viable cells. Reverse-transfected Caco-2 and HTC116 cells were seeded in a 96-well plate (5 × 10^3^ and 1 × 10^4^ cells per well, respectively). After 72 h of cultivation, the MTT assay was performed as described previously [19]. Light absorbance was detected with a Sunrise plate reader (Tecan, Männedorf, Switzerland) at a wavelength of 570 nm, with a reference wavelength of 620 nm. At least three independent experiments were performed.

### 4.8. Wound Healing Assay

Reverse-transfected Caco-2 and HTC116 cells were seeded in a 2-well silicone insert with a defined cell-free gap (Ibidi, Gräfelfing, Germany) for the wound healing assay (4.5 × 10^4^ and 6 × 10^4^ cells per well, respectively) and cultured until they reached 90% confluency. After the insert’s removal, the formed gap was captured at four different time points every 24 h with an inverted light microscope (Olympus IX71, Tokyo, Japan). The ratio between the remaining and the initial size of the wound area was evaluated using the ImageJ software (version 1.54f) (Bethesda, MD, USA). At least three independent experiments were performed.

### 4.9. Clonogenic Assay

Reverse-transfected HCT116 and SW620 cell lines were plated at a density of 250 cells per well within a 6-well culture plate. Following a two-week incubation period in a complete growth medium, cells underwent sequential treatment steps, including PBS (Gibco, Waltham, MA, USA) washing, fixation with 10% formaldehyde (Sigma Aldrich, Burlington, MA, USA) for 20 minutes, and subsequent staining with 1% crystal violet (Alfa Aesar by Thermo Fisher Scientific, Kandel, Germany) for 15 minutes at ambient temperature. Quantification of colony-forming units was performed utilizing ImageJ software (version 1.54f) (Bethesda, MD, USA), leveraging data acquired from three distinct experimental replicates.

### 4.10. Cell Proliferation Assay

Reverse-transfected HTC116 and SW620 cells were seeded in 6-well plates (2 × 10^5^ cells per well). After 24 h, cells were stained using the CellTrace CFSE Cell Proliferation Kit (Thermo Fisher Scientific, Waltham, MA, USA) according to the manufacturer’s recommendations, as described in a previous publication [76]. Cells treated with inhibitor-NC were analyzed at 0 h and 96 h, whereas cells treated with the isomiR inhibitors were analyzed at 96 h with BD FACSMelody (BD, New York, NY, USA), using 488 nm excitation and emission filters.

### 4.11. Apoptosis Assay

Apoptosis and necrosis tests were performed using the Annexin V Pacific Blue™ conjugate for flow cytometry (Thermo Fisher Scientific, Waltham, MA, USA), 72 h post-transfection with miRNA inhibitors, according to the manufacturer’s protocol, as described in our previous study [76]. Samples were analyzed using a FACSMelody™ Cell Sorter (BD Biosciences, New York, NY, USA).

### 4.12. Statistical Analysis of Loss of Function Experiments

All data from the functional experiments in the CRC cell lines are given as the means ± standard errors (SE) of at least three independent experiments. Data between groups were compared using a two-tailed Student’s t-test for normally distributed data or the Mann–Whitney U test for non-normally distributed data (shown via the Shapiro–Wilk test). All statistical calculations were performed with the R software version v3.6.0 (Boston, MA, USA). A false discovery rate (FDR) corrected *p* < 0.05 was considered statistically significant.

## 5. Conclusions

The transcriptome analysis revealed that WT-miR-1246 and its two isoforms can dysregulate a wide range of genes that are potentially involved in carcinogenesis. A number of genes that are predicted to be targets of the miRNAs in silico were found to be downregulated in our transcriptome study (e.g., *EREG*, *LATS2*, *PPP2CB*). Among these were genes that are known to affect apoptotic processes, migration, and proliferation and cause genetic instability. The pathway enrichment analysis indicated that cancer-related pathways tend to be more dysregulated in the case of ISO-miR-1246_a and ISO-miR-1246_G (PI3K/AKT signaling and, most notably, Hippo), whereas cell cycle pathways were more dysregulated in WT-miR-1246. The most carcinogenic downregulated targets of miR-1246 and its isoforms were *LATS2*, *EREG*, and *PPP2CB*. The loss of function cell experiments consolidated these expression results further, as WT-miR-1246 and its isoforms affected colorectal cells’ viability, colony formation rate, migration, and apoptosis.

## Data Availability

The raw sequencing data, as well as miRNA counts, have been deposited at the Gene Expression Omnibus (GEO) under the accession number GSE237144. All other data presented in this study are included within the paper and its Supplementary Information or are available upon request from the corresponding author.

## Supplementary Materials

The following are available online at https://www.mdpi.com/article/10.3390/ijms25052808/s1. References [60,61,72,73,74,77,78,79,80,81,82,83] are cited in the supplementary materials.
