# Supplementary material for: 5′-Isoforms of miR-1246 Have Distinct Targets and Stronger Functional Impact Compared with Canonical miR-1246 in Colorectal Cancer Cells In Vitro
Source: Int J Mol Sci. 2024 Feb 28;25(5):2808. doi: 10.3390/ijms25052808 (PMC10931981; doi:10.3390/ijms25052808)
Supplement: Supplementary file 1 [file ijms-25-02808-s001.zip › ijms-2832391-supplementary.pdf]

SUPPLEMENTARY MATERIALS

## miRNA transcriptome of Caco-2

As a model cell line to test impact of 5’ miR-1246 impact on expression of protein coding genes the Caco-2 cell line was used. To assess the natural level of miR-1246 expression the miRNA sequencing was conducted. The results are available in supplementary figure 1.

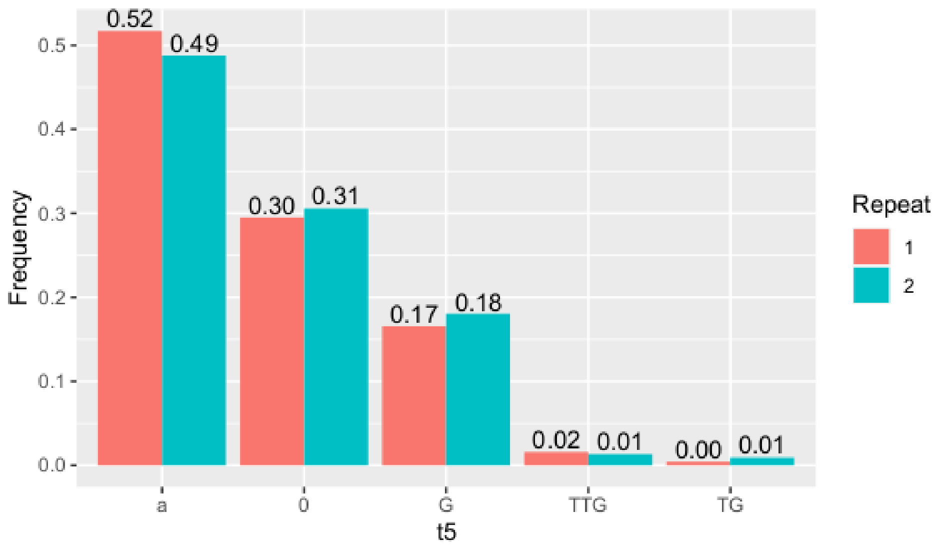


**Supplementary Figure S1.** Relative abundance of 5’ isoforms of miR-1246 in Caco-2 adenocarcinoma cells. The figure indicates the frequency of 5’miR-1246 modifications. The x-axis denotes 5’ modification (t5), the y-axis denotes the modifications frequency, the colors denotes a repeat.

The analysis was done as follows: adapter were trimmed-of and quality trimming was done using Trim Galore v 0.6.10 with these flags “-fastqc --small_rna --length 17 --max_length 40“. miRNAs/isomiRs in the fastq sequences were annotated using miraligner from seqbuster v3.5 with these flags: "-sub 1 -trim 3 -add 3 -s hsa -freq". As a reference - dataset tagged as “CURRENT” were used from miRBase (https://www.mirbase.org/ftp/CURRENT, downloaded at June2023). The sequencing data is available at GEO under the accession number of GSE237144 (secure token: ghafwssortotnmj).

In total miR-1246 variants make up 0.155 % (average of two repeats) of all miRNAs. As we see in supplementary figure 1 (lower figures) there are three most frequent 5’ isoforms. The most frequent one is removal of “A”, followed by intact 5’ end and addition of one “G”. As for the 3’ modifications – the analysis reveals that two variants are the most common: extension by “A” or the intact variant.

## Overlap of gene sets matching predicted targets of mi R-1246 isoforms

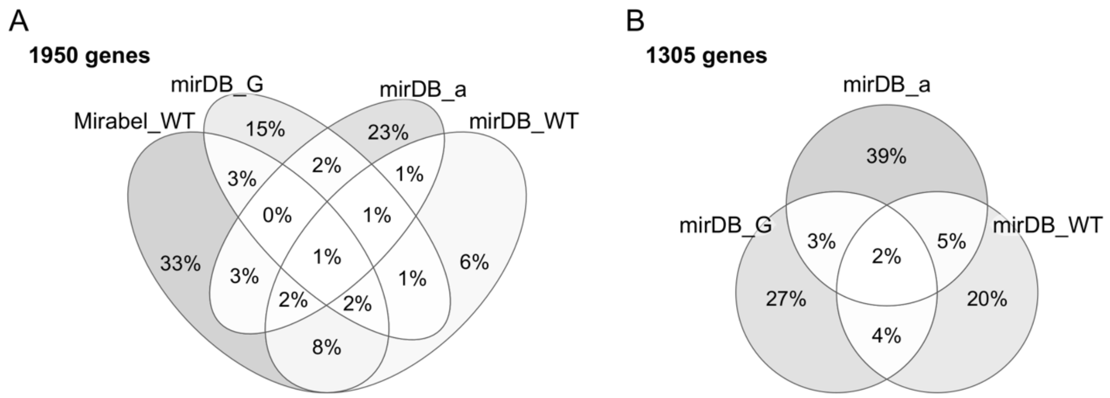


**Supplementary Figure S2.** Overlap of gene sets matching predicted targets of miR-1246 isoforms The set labels indicate the database and isoform that was used for predictions (i.e., Mirabel_WT and mirDB_WT denote predictions for the canonical sequence from the mirDB and Mirabel databases. Suffix "_a" denotes ISO-miR-1246_a, suffix "_G" – ISO-miR-1246_G). The intersection color denotes the matching count number: the higher the number is, the darker tone is. The left panel (A) denotes intersections among three predictions by mirDB (one for each miR-1246 isoform) and targets predicted by Mirabel for the wild type canonical variant. The right panel (B) depicts intersections among datasets derived from mirDB only.

## Choice of enrichment methods

At first analysis of impacted biochemical pathways was done analyzing differential analysis results for the WT-miR-1246 (gene counts of samples affected by WT-miR-1246 comparing to the ones affected with miRNA mimic negative control). The results on the number and statistical significance on the impacted pathways analyzing the Reactome and KEGG pathways are given in the **Supplementary Figures S3** and S**4** respectively. Seven different topology-based algorithms were tested. As it is evident in the **D** and **E** parts of the **Supplementary Figures S3-S4** some algorithms indicate a lot of pathways as being significantly impacted. In case of the three algorithms NETGSA [81], Pathway-Express [82] and NEAT [83] more than half of all P-values (corrected for multiple comparisons) were less than 0.05. On the other hand, the DEGraph [84] as implemented in the EnrichmentBrowser RELEASE_3_13 [85] for a significant fraction of pathways failed to finish calculations as it is depicted in the **A** parts of **Supplementary Figures S3** and S**4**. Finally, we chose to use two methods that indicated the smallest number of statistically significantly impacted pathways: GGEA [34] and PathNet [35]. *Ma et all*. states [86] DEGraph is the most robust, followed by PathNet. As DeGraph failed to calculate P-values for many pathways, we chose combination of PathNet [35] and GGEA [34]. As it is evident in the **C** and **D** parts of **Supplementary Figures S3** and S**4**, GGEA results are most similar to the PathNet results apart from DeGraph. The final ranking of the pathways in terms of dysregulation was based on the combined results of PathNet and GGEA results.

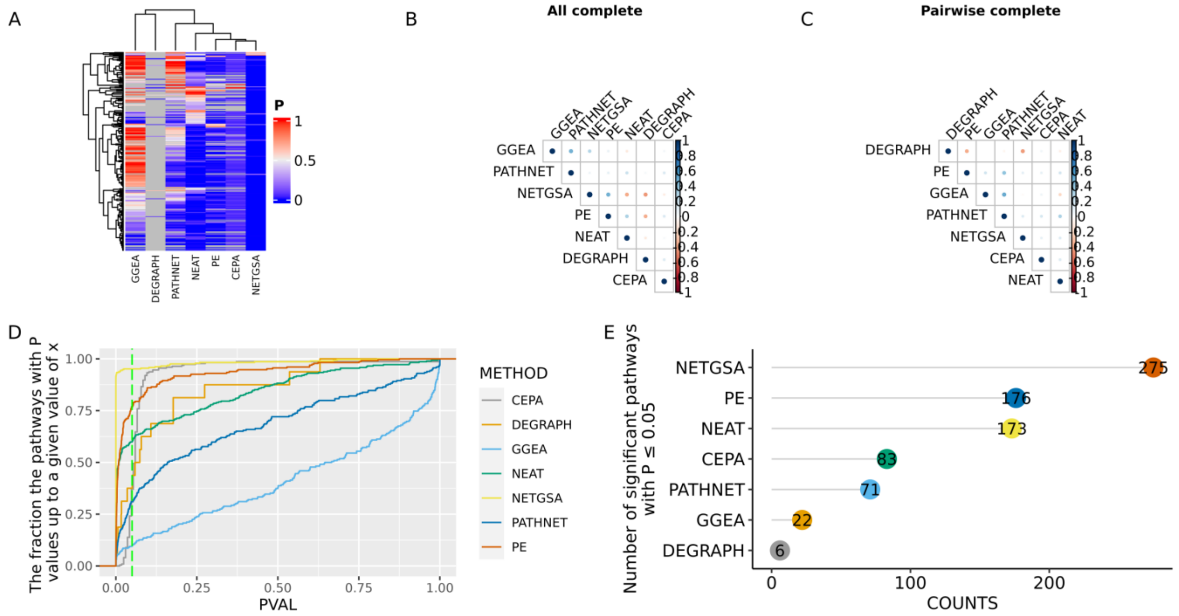


**Supplementary Figure S3.** Comparison of prediction efficacy/indicators of enrichment methods for the gene sets from the KEGG database. Seven different enrichment methods were tested using one set of DE data (mimic group of WT-miR-1246). **A -** the P-values for being impacted for each KEGG gene set. The gray color indicates that calculation by the algorithm failed. The clustering trees are based on Euclidean distance. **B -** pairwise correlations between impact ranks based on P-values. Only gene sets having calculated values for all the methods were considered (all complete). **C -** pairwise correlations between impact ranks based on P- values. Only gene sets having calculated values for the two methods being compared were considered (pairwise complete). The green vertical line marks P-value of 0.05. **D -** Empirical cumulative distribution of the P-values. **E -** number of enrichment pathways that were significantly impacted.

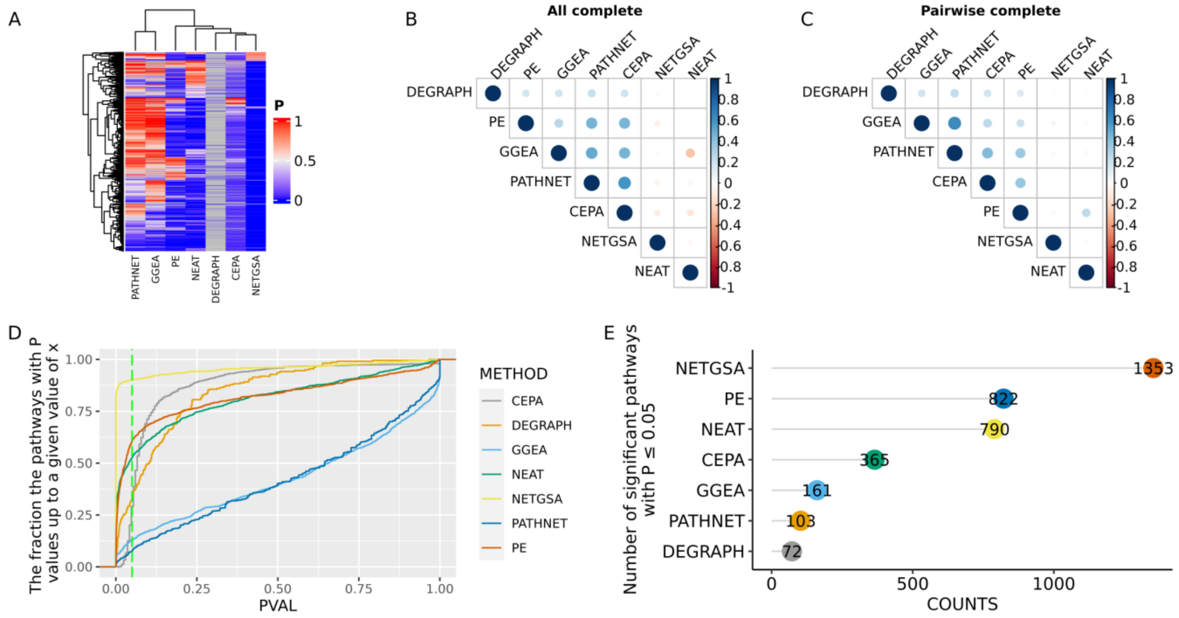


**Supplementary Figure S4**. Comparison of enrichment methods results for the gene sets from the Reactome database. Seven different enrichment methods were tested using one set of DE data (mimic group of WT-miR-1246). **A -** the P-values for being impacted for each Reactome gene set. The gray color

indicates that calculation by the algorithm failed. The clustering trees are based on Euclidean distance. **B -** pairwise correlations between impact ranks based on P-values. Only gene sets having calculated values for all the methods were considered (all complete). **C -** pairwise correlations between impact ranks based on P-values. Only gene sets having calculated values for the two methods being compared were considered (pairwise complete). The green vertical line marks P-value of 0.05. **D -** Empirical cumulative distribution of the P-values. **E -** number of enrichment pathways that were significantly impacted.

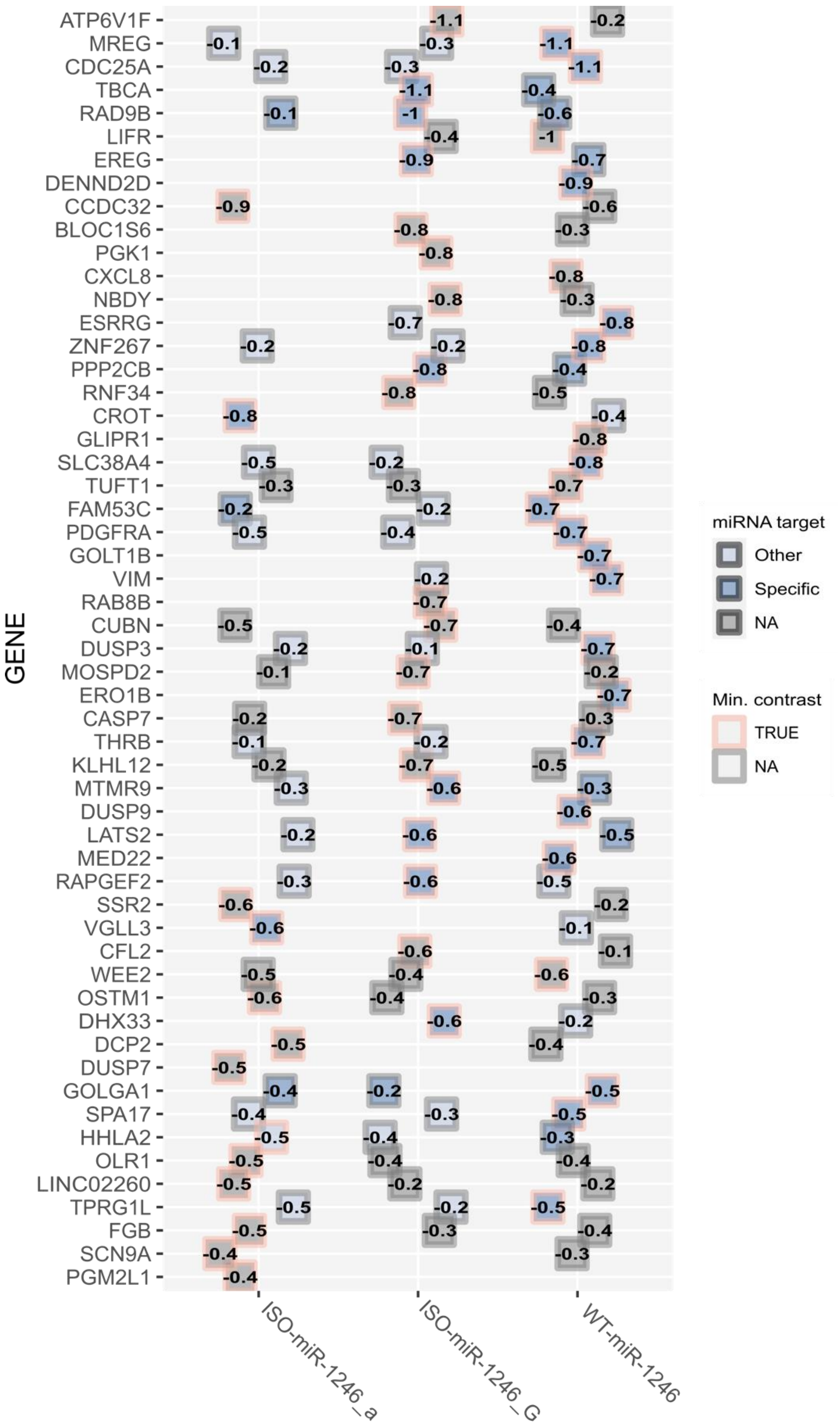


**Supplementary Figure S5.** The 20 top most downregulated genes for each mimic group. The absolute values of the $log_2FC$ are indicated by the black labels. The labels in color indicate the most significantly impacted Reactome pathway that includes a particular gene. The fill of the square indicates if a gene is a predicted target for a specific (corresponding) isomiR variant. The blue color indicates that a particular gene is predicted to be a target of the corresponding miRNA. The pale blue color indicates that a particular gene is predicted to be a target of a miRNA variant other than the one matching the corresponding mimic group. The genes are arranged from the top to bottom based on the absolute value of the $log_2FC$. The pinkish-orange color indicates the group with the minimum $log_2FC$ for a particular gene.

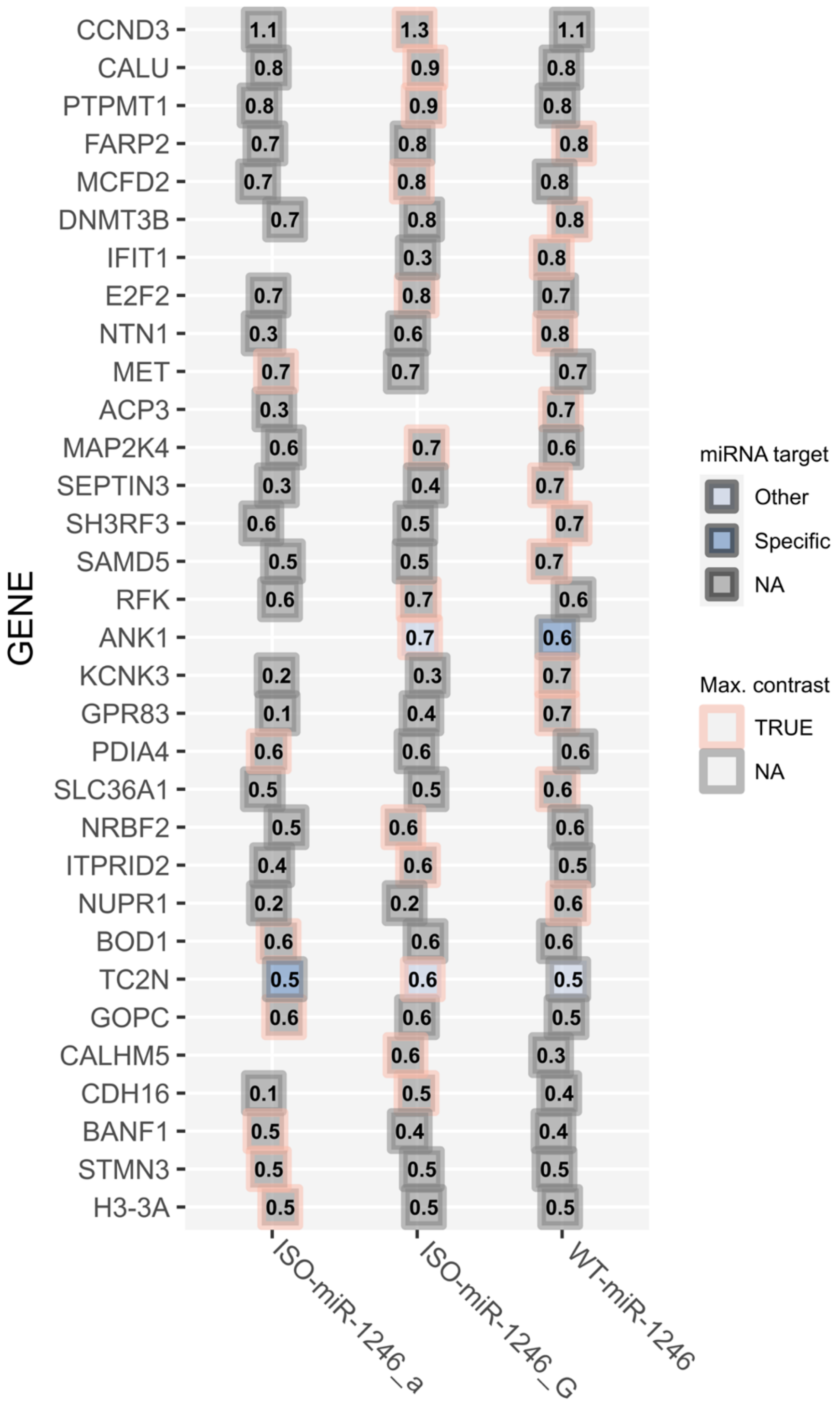


**Supplementary Figure S6.** The 20 top most upregulated genes for each mimic group. The absolute values of the $log_2FC$ are indicated by the black labels. The labels in color indicate the most significantly impacted Reactome pathway that includes a particular gene. The fill of the square indicates if a gene is a predicted target for a specific (corresponding) isomiR variant. The blue color indicated that a particular gene is predicted to be a target of the corresponding miRNA. The pale blue color indicates that a particular gene is predicted to be a target of a miRNA variant other than the one matching the corresponding mimic group. The genes are arranged from top to the bottom based on the absolute value of the $log_2FC$. The pinkish-orange color indicates the contrast with the maximum $log_2FC$ for a particular gene.

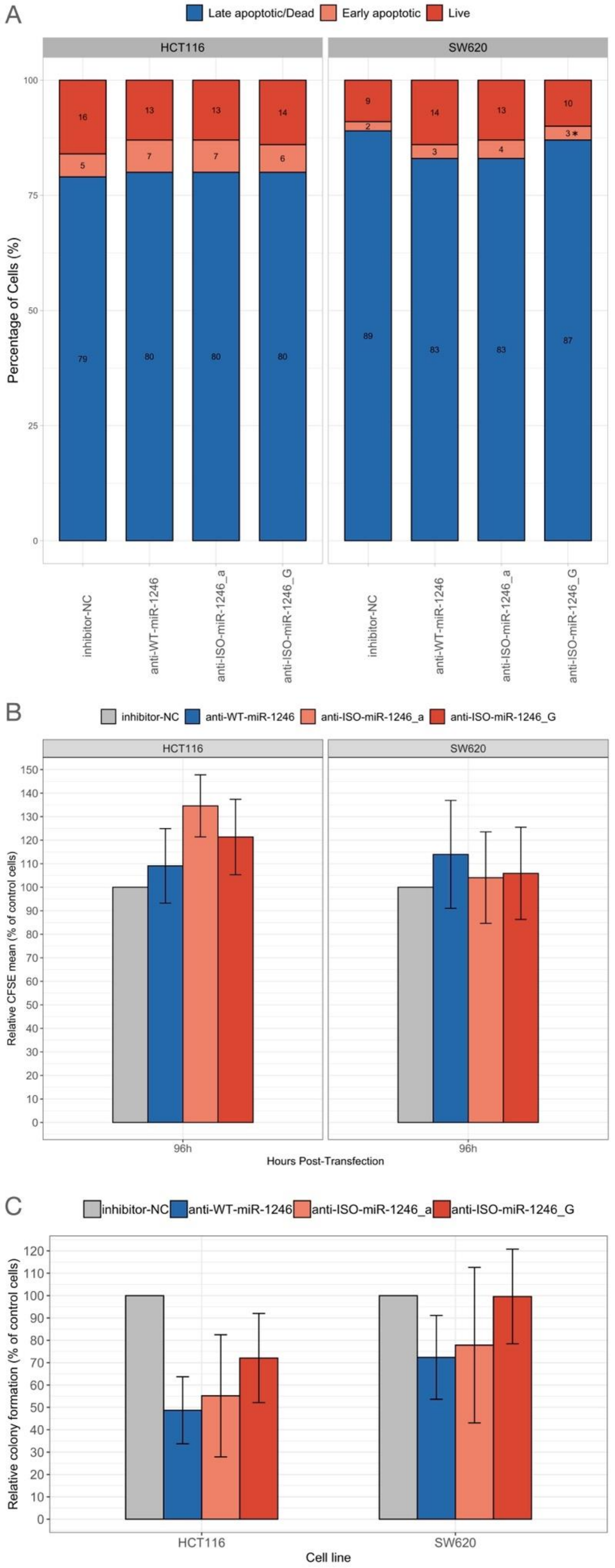
A
Late apoptotic/Dead
Early apoptotic
Live
HCT116
SW620
Percentage of Cells (%)
100
75
50
25
0
16
5
79
13
7
80
13
7
80
14
6
80
9
2
89
14
3
83
13
4
83
10
3*
87
inhibitor-NC
anti-WT-miR-1246
anti-ISO-miR-1246_a
anti-ISO-miR-1246_G
B
inhibitor-NC
anti-WT-miR-1246
anti-ISO-miR-1246_a
anti-ISO-miR-1246_G
HCT116
SW620
Relative CFSE mean (% of control cells)
150
140
130
120
110
100
90
80
70
60
50
40
30
20
10
0
96h
Hours Post-Transfection
C
inhibitor-NC
anti-WT-miR-1246
anti-ISO-miR-1246_a
anti-ISO-miR-1246_G
Relative colony formation (% of control cells)
120
110
100
90
80
70
60
50
40
30
20
10
0
HCT116
SW620
Cell line

**Supplementary Figure S7. A -** barplot represents relative colony formation (% of control cells) in SW620 and HCT116 cells after inhibition of miR-1246 and its isoforms. A tendency of reduced formation of colonies was observed in both SW620 and HCT116 cells compared to inhibitor negative control (inhibitor-NC). **B -** barplots represents relative CFSE (% of control cells) in SW620 and HCT116 cells after inhibition of miR-1246 and its isoforms. A tendency of slowed proliferation was observed in both SW620 and HCT116. **C** – Stacked barplot represents the percentage of live, early apoptotic, or late apoptotic/dead cells 72h after transfection with miRNA inhibitors. Data are presented as the mean (three or more independent experiments) ± standard error (SE), * – data is statistically significant when $P < 0.05$. Results are shown as units relative to the mimic negative control (inhibitor-NC).

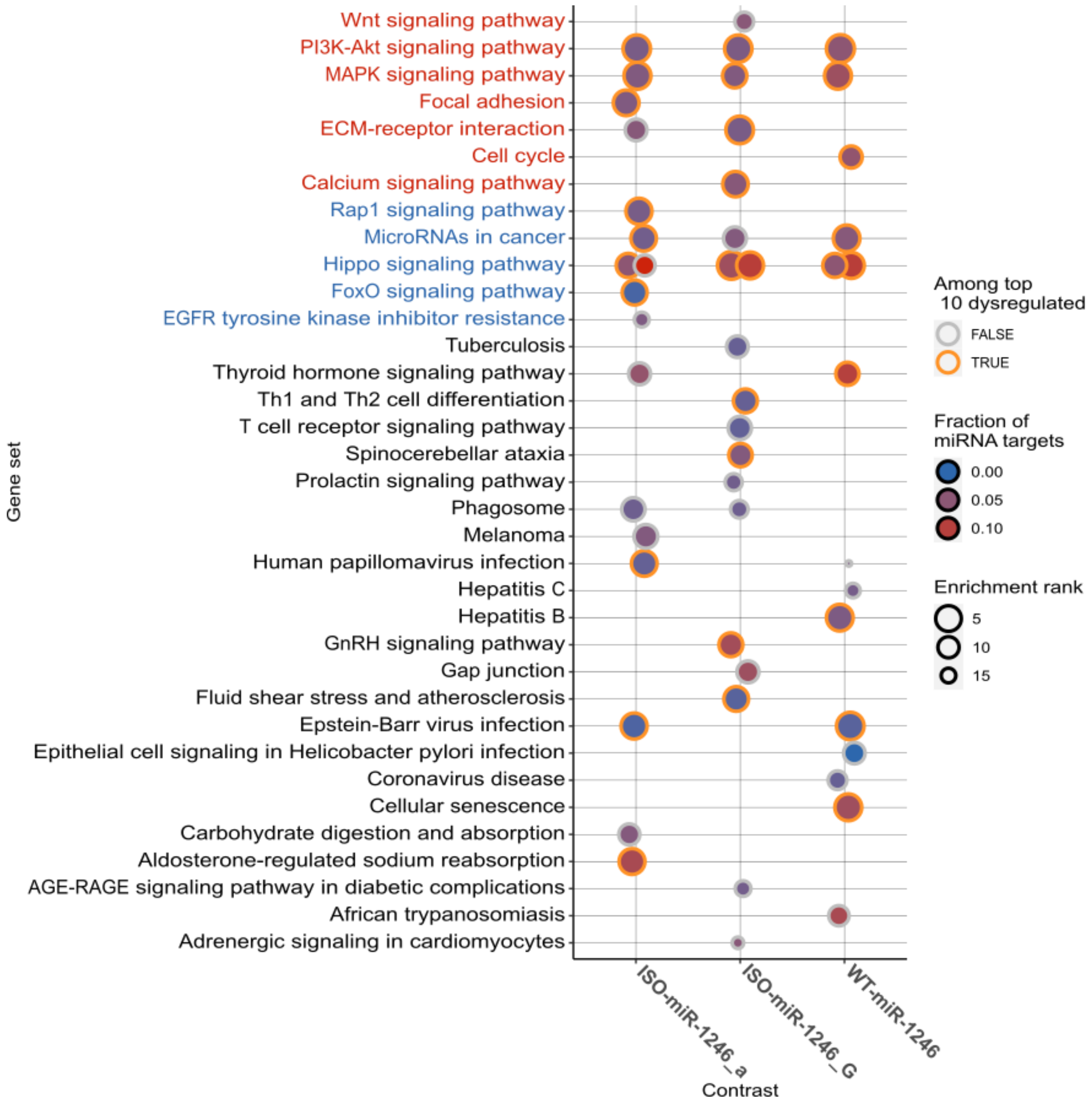


**Supplementary Figure S8**. The top enriched pathways for KEGG database. For each mimic group the top most enriched pathways were chosen for visualization. The pathways that are matched to subpathways of KEGG cancer pathways (hsa05200) are denoted by the red label color. The blue color indicates other potential cancer related pathways. The circle size is inversely proportional to the pathway enrichment rank, i.e., the larger the circle, the more significant enrichment. If a gene set is among the top 10 gene sets for a particular mimic group, the border of the corresponding circle is orange. A double bubble at the Hippo signaling pathway is due to the fact that the KEGG database contains two pathways named under Hippo (hsa04392 and hsa04390).

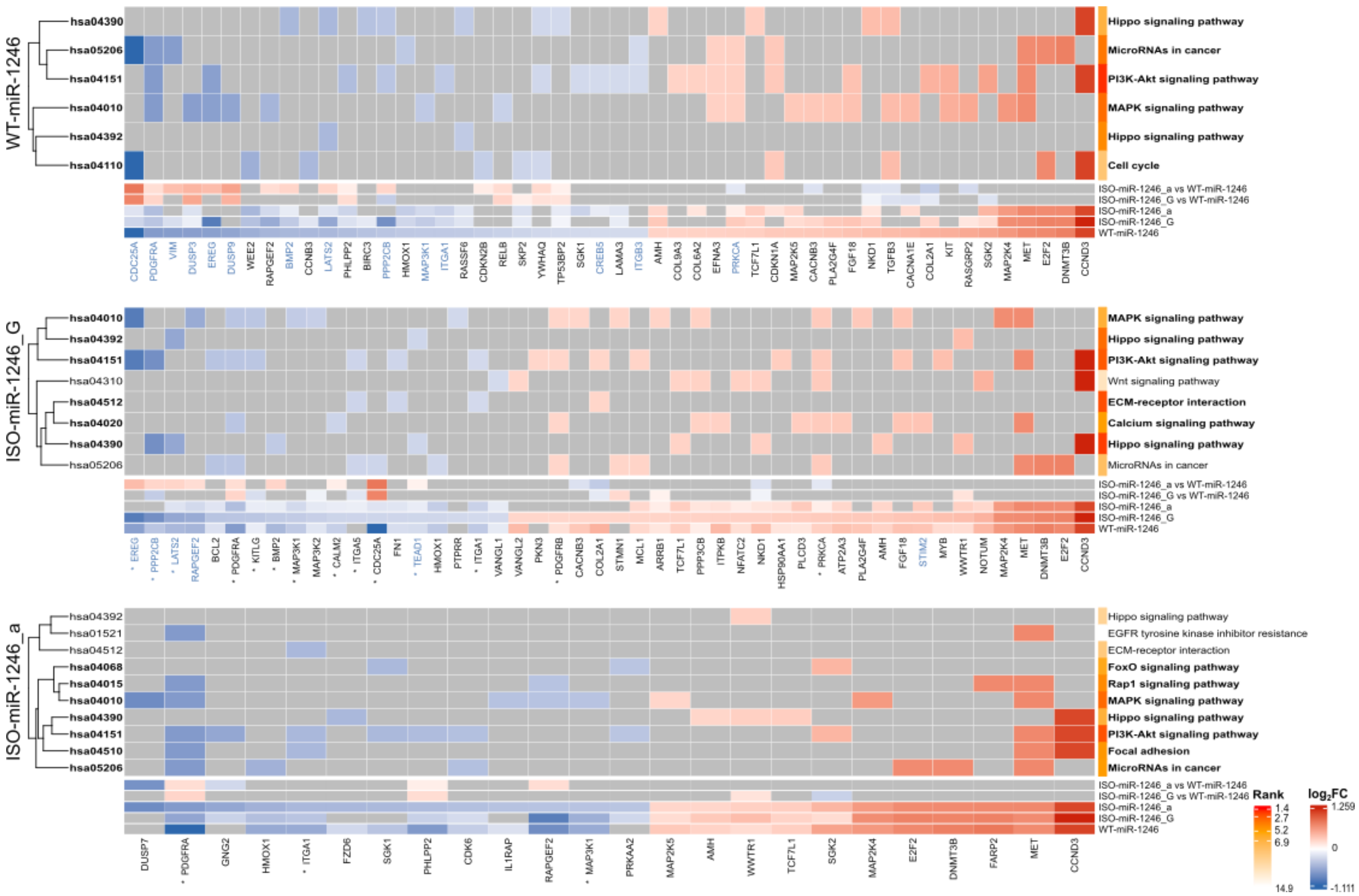


**Supplementary Figure S9.** The most differentially expressed genes matching mimic group comparing miRNA mimic with the negative control for the cancer related KEGG pathways. The three mimic groups are indicated at the left of each heatmap. Only pathways that are found to be significantly enriched by the two programs (PathNet and GGEA) are shown for each group. Pathway ids are given by the left side of the row, the descriptions - by the right. If a pathway is in the top 10 enriched pathways for a mimic group, its description is given in bold font. The mean significance rank is indicated by the color gradient in the right annotation heatmap from red to orange with red indicating the highest significance and the lowest rank. The genes indicated as column names are those with the corresponding absolute value of $\log_2$FC is $\geq 0.25$. The grey color in the heatmap indicates that a gene doesn't belong to a pathway. The blue color of a gene name indicates that the gene is predicted to be a target of a specific mimic group. In the case of the isoforms the asterisk by the gene names marks genes that were predicted to be targets of

WT-miR-1246 either by MirDB or Mirabel. The heatmaps at the bottom indicate the expression level of the genes across all explored groups with grey color indicating insignificant differential expression.

## Analysis of silencers

To test if gene expression can be related to the suppression of the matching silencer (targets of miRNA) by the miRNAs the data from SilencerDB [87] (downloaded 2022 04 16) was analyzed. For the validated silencers all datasets were used and for the predicted silencers the data subset to match intestine, liver, pancreas, skin, stomach, and stem cells organs were used. The results are given in the **Supplementary Figure S10**.

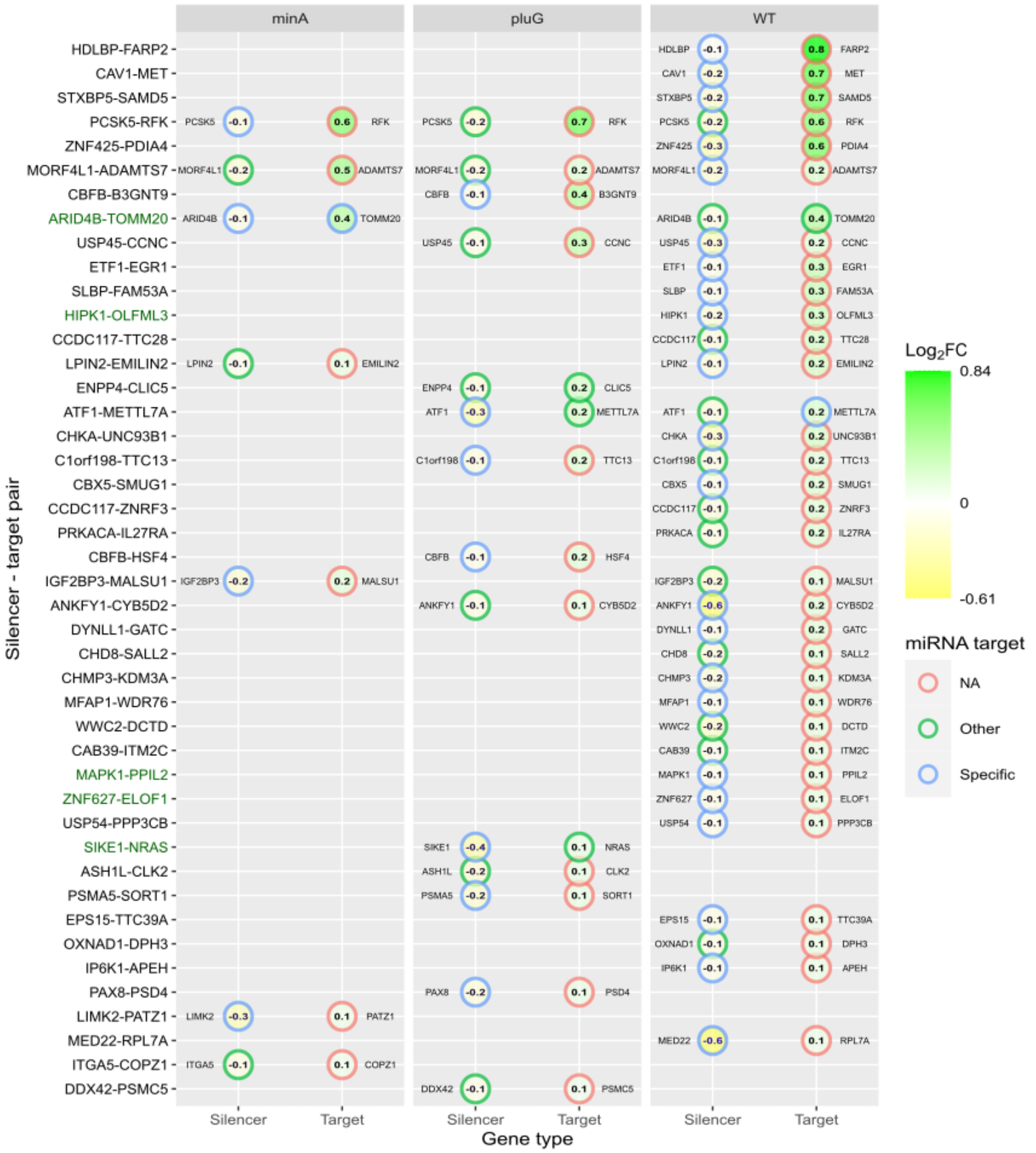


**Supplementary Figure S10**. miRNA targeted gene silencers. For each mimic group the known gene silencer targeted by the miRNAs are indicated. Only pairs with decreased expression of a silencer and increased expression of a target are shown. The circle size is proportional to the *absolute* value of the $log_2FC$, which is indicated by black labels. Y axis' labels indicate silencer - target pairs. Labels for the validated pairs are denoted in green, predicted - in black. The labels by the circles' sides indicate names of the particular genes. The X axis' labels indicate if a gene is a silencer or a target. The fill of the circle indicates if a gene is a predicted target for a corresponding miR-1246 isoform. Cyan color indicated that a particular gene is predicted to be a target of the corresponding miRNA (indicated on the top of the panels),

pinkish-orange color indicates that a particular gene is predicted to be a target of a miRNA variant other than the one matching the corresponding mimic group. The genes are arranged from top to bottom based on the absolute value of the $log_2$ of the targets' fold changes.

## Analysis of immunoprecipitation data from other studies

We did an extensive literature/available public data analysis to elucidate if miR-1246 indeed can form complexes with AGO proteins. The compiled immunoprecipitations data related to miR-1246 from several published studies is available in supplementary figure 11.

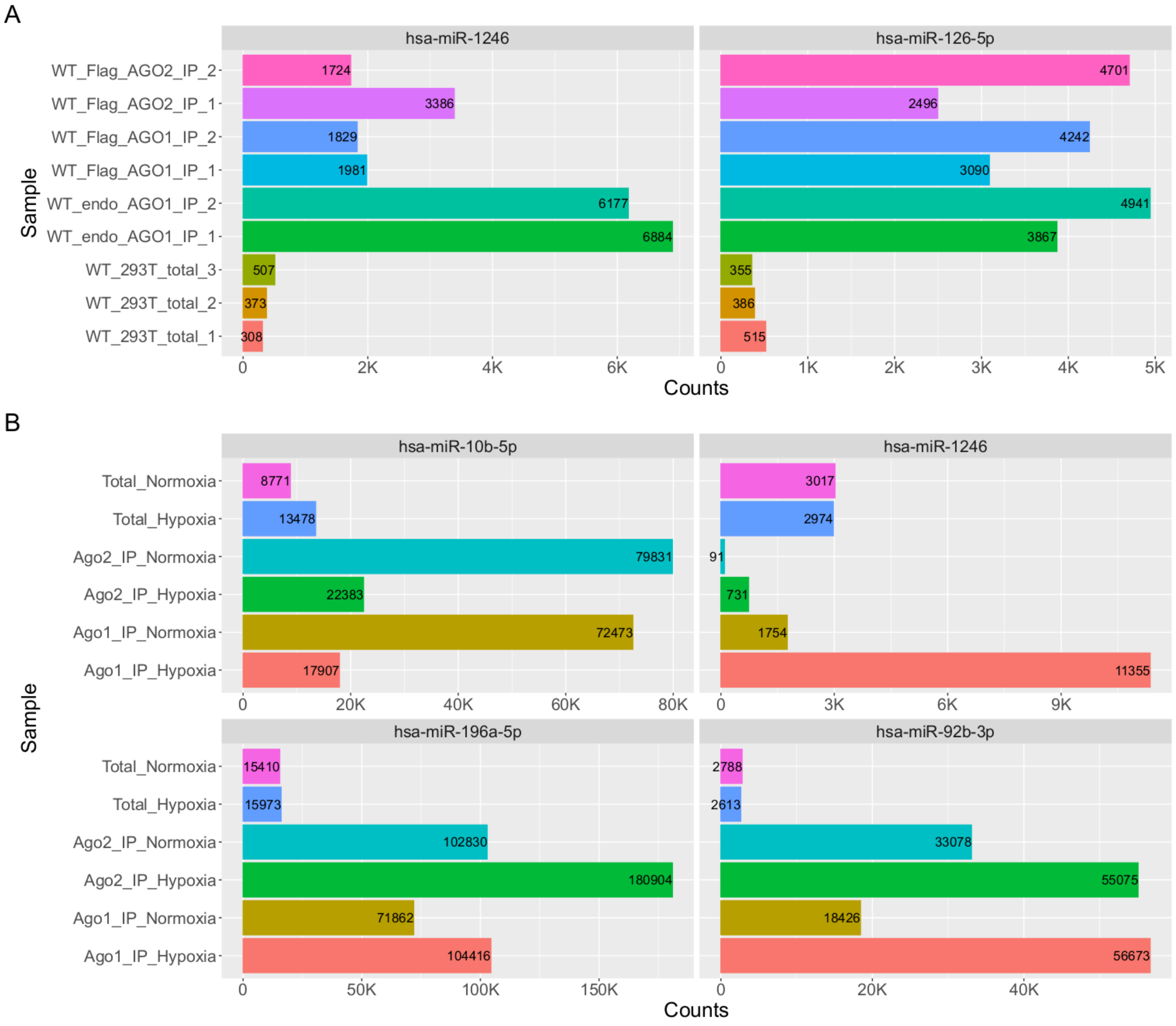


**Supplementary Figure S11**. Immunoprecipitation data on miR-1246 showing association with Ago proteins from other studies. Some other miRNAs mentioned in the associated articles are also showed in addition to the miR-1246 (denoted as "hsa-niR-1246"). A, data from the NCBI BioProject PRJNA840916. B, data from the NCBI BioProject PRJDB2698. The values along the Y-axis indicate noramlised by DESeq2 counts. The values along the Y-axis represent associated BopSample's title with the fragment "_replicate" removed.

The data from NCBI BioProject PRJDB2698 [PMID: 23939471] was obtained from the following SRA entries: DRS001636, DRS001637, DRS001638, DRS001639, DRS001640, and DRS001641. The reads processed using the nf-core/smrnaseq v2.0.0 workflow [citavimui nurodo sita linka https://zenodo.org/record/7930043]. The reads before analysis with the workflow were trimmed using BBduk program from BBtools v38.87 package [28]. In the article

[PMID: 23939471] the sequencing kit used is not indicated and sequences for trimming were empirically selected, these are: ATCTCGTATGCCGT, TATCTCGTATGCCG, CATCTCGTATGCCG, TCTCGTATGCCGTC, AATCTCGTATGCCG. The counts were taken from the miRTop output file (“mirtop_rawData.tsv”) and further normalized using DESeq2 v 1.38.0. The data for analysis in the study NCBI BioProject PRJNA840916 [PMID: 36071058] was derived from the corresponding miRNA counts available for download in the associated data files from the related GEO entries. The counts from the following SRA entries were utilized: SRR19333132, SRR19333131, SRR19333130, SRR19333129, SRR19333128, SRR19333127, SRR19333126, SRR19333124, and SRR19333122." They were further normalized using DESeq2 v 1.38.0.

As we see, miR-1246 is enriched when immunoprecipitation is done using AGO1 antibodies in two studies (Supplementary figure 11, A and B) and in one study (Supplementary figure 11, A) when AGO2 based immunoprecipitation is used. Therefore, other studies support our claim that miRNA-1246 acts as a miRNA and forms complexes with AGO proteins.

**Enrichment of downregulated miRNA targets after transfection**

We have assessed whether the predicted miRNA targets are over-represented among the down-regulated genes after transfection with mimics – the data is available in supplementary figure 12.

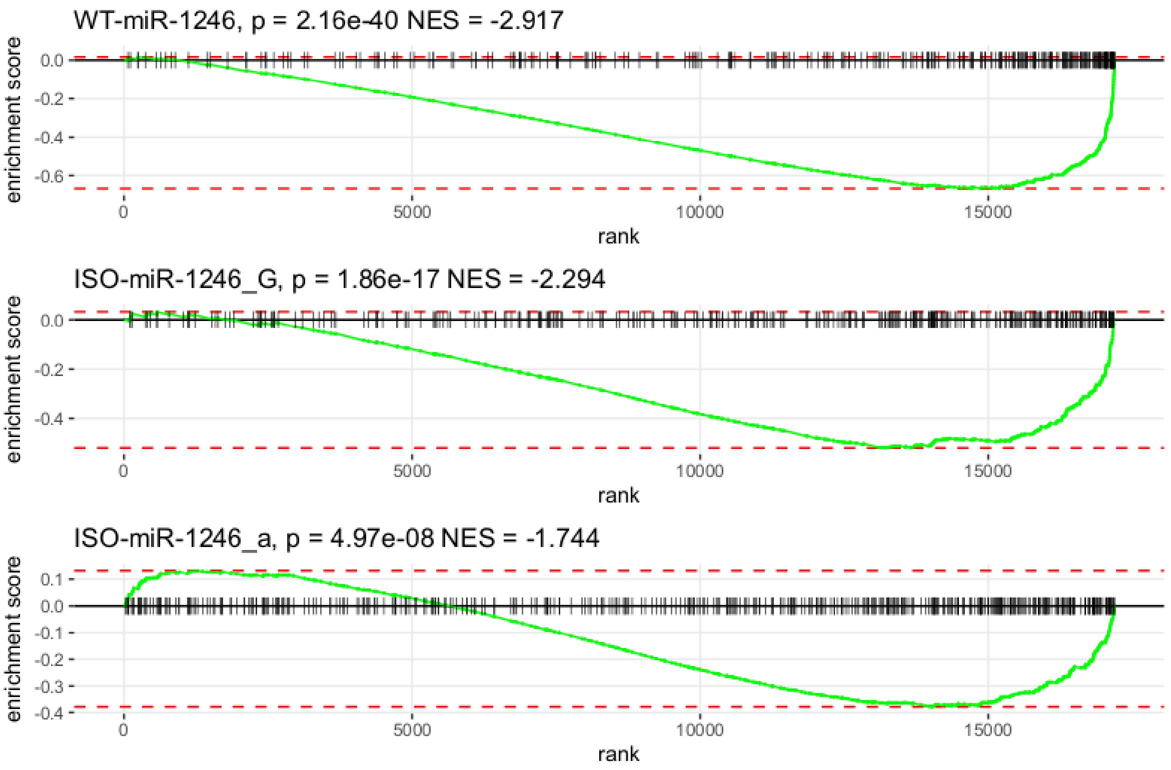


**Supplementary Figure S12**. Test of enrichment of miRNA targets among the downregulated genes. The enrichment plots from top to bottom match data for WT-miR-1246, ISO-miR-1246_G, ISO-miR_a variants correspondingly. The black vertical dashes along the X-axis depict genes that were indicated as miRNA targets, X-axis matches genes that are ranked from the most positive $\log_2$ of the genes (the

leftmost side) fold changes to the most negative ones (the rightmost side). The green curves depict matching enrichment scores.

The enrichment of miRNA targets among the downregulated genes was tested using FGSEA v 3.16 [DOI: 10.18129/B9.bioc.fgsea] The genes before enrichment test were sorted by their log2 fold changes using the mimic negative control as a reference (please look for details on the differential expression calculations in the main text). The genes that are potential miRNA targets were retrieved from publicly available database miRDB.org v6.0 [32] - a specific set for the miRNA sequence variants were used for each sequence variant.

As we see from the p values (supplementary figure 12) the most significant enrichment is detected in the case of WT-mi-1246 ($p = 2.16e-40$) with Normalized Enrichment Score (NES) equal to –2.917. A bit less significant enrichment is detected in the case of ISO-miR_G ($p = 1.86e-17$, NES = -2.294). Not so strong enrichment albeit still significant is detected for ISO-miR-1246_a ($p = 4.97e-08$, NES = -1.744). Therefore, the genes having potential targets of the miRNAs tend to be downregulated as expected. Apart from the statistical value this is visually evident looking at the distribution of genes with predicted targets among the list sorted from most upregulated to the most downregulated (supplementary figure 12, vertical black dashes along the X-axis indicates the most downregulated genes that in the rightmost side)
